# *Ilex paraguariensis* extracts extend the lifespan of *Drosophila melanogaster* fed a high-fat diet

**DOI:** 10.1590/1414-431X20176784

**Published:** 2017-11-30

**Authors:** A.C. Colpo, M.E. Lima, H.S. da Rosa, A.P. Leal, C.C. Colares, A.C. Zago, A.C.F. Salgueiro, P.R. Bertelli, L. Minetto, S. Moura, A.S.L. Mendez, V. Folmer

**Affiliations:** 1 Programa de Pós-Graduação em Bioquímica, Universidade Federal do Pampa, Uruguaiana, RS, Brasil; 2 Laboratório Escola de Análises Clínicas, Curso de Farmácia, Universidade da Região da Campanha, Bagé, RS, Brasil; 3 Faculdade de Farmácia, Universidade Federal do Rio Grande do Sul, Porto Alegre, RS, Brasil; 4 Laboratório de Biotecnologia de Produtos Naturais e Sintéticos, Instituto de Biotecnologia, Universidade de Caxias do Sul, Caxias do Sul, RS, Brasil

**Keywords:** Yerba mate, Matesaponin, LC-MS, Antioxidants compounds, Chronic diseases, Oxidative stress

## Abstract

Studies have suggested that total energy intake and diet composition affect lifespan and ageing. A high-fat diet induces oxidative stress and affects the development of diseases. In contrast, antioxidants are capable of reducing its harmful effects. Yerba mate beverages are an important source of antioxidants, but there is scarce knowledge about their effects on suppressing fat accumulation. Here, we investigated the compounds present in yerba mate extracts and assessed their effects on *Drosophila melanogaster* given a high cholesterol diet. LS-ESI-MS analysis showed the presence of matesaponins, phenolic compounds and methylxanthines in all of the examined extracts. In *Drosophila,* under extract treatment conditions, the mean lifespan was significantly extended from 38 to 43 days, there was an increase in the ability to support induced stress and decrease in lipid peroxidation products. Moreover, yerba mate extracts recovered the glutathione S-transferases (GST) activity and reduced the cholesterol level. Taken together, our results support that extracts can extend lifespan by reducing the detrimental effect of a high-fat diet in *D. melanogaster,* and this outcome can be associated with the compound content in the extracts. This study improves the understanding of natural interventions that reduce stress-induced oxidative damage, which is fundamental in promoting healthy ageing.

## Introduction

Diets should ideally have a nutrient balance. This is an important determinant of fitness and lifespan in living organisms. Both caloric restriction and natural compounds in the diet extend the lifespan and delay the occurrence of age-related diseases in various aging models (1).

In contrast, the level of reactive oxygen species (ROS) produced by mitochondrial metabolism increases during excessive food intake (2), which reduces the lifespan and increases the risk of disease (3). A high-fat diet can induce obesity, and adipose tissue generates reactive species (RS) (4,5).

In this setting, compounds with antioxidant properties can alleviate the deterioration caused by ROS and delay the aging process (5). Polyphenols may play protective roles through the hydrogen atom transfer, single electron transfer and metal chelation (6). Moreover, Ríos-Hoyo et al. (7) observed that polyphenols can evoke beneficial effects by exerting antioxidant activity and acting through metabolic pathways that enhance cardiovascular health, promoting vasodilatory, anti-atherogenic, antithrombotic, and anti-inflammatory effects. Saponins, in turn, induce reduction of the fat weight, plasma triglyceride level, and appetite as well as inhibit pancreatic lipase (8).

*Ilex paraguariensis* extracts are an important source of polyphenols, methylxanthines (9) and saponins (8). Many studies describe their effect on protecting against ROS and stimulating cells' antioxidant defenses (9,10). Traditionally, yerba mate beverages are consumed in Brazil, Argentina, Uruguay, and Paraguay, and are named "Chimarrão", "Mate" or "Tererê" (11). Mate consumption occurs with sequential extractions. However, few groups have been working on analyzing the extracts obtained with this approach.

*Drosophila melanogaster* is considered a good pragmatic model for evaluating metabolic disorders. Fruit flies have many of the same basic metabolic functions as mammals, including the ability to maintain glucose homeostasis, store and mobilize energy, and modulate food intake, and many molecular mechanisms that regulate these metabolic processes are conserved in this model (12).

In this study, using extracts obtained from yerba mate, we evaluated the presence of different compounds with antioxidant potential. We also explored the capacity of extracts to reduce fat accumulation, improving stress resistance and extending the lifespan of fruit flies that were fed a high cholesterol diet.

## Material and Methods

### Extract preparation

Aqueous extracts were obtained by recreating the traditional mate preparation process. We used yerba Baldo¯, a brand marketed in Uruguay. Mate was prepared in a medium-size gourd; the yerba mate level occupied two thirds of the volume in the bowl (85 g). The remaining volume was filled with water (70 mL) at 80°C. After 1 min the water was removed through a pump attached to a suction system. The sequential infusion (mate) extracts 1, 2, 5, 10, and 15 were stored for further analysis, and all other extractions were discarded. After the extraction, mate extracts were filtered using filter paper (thickness 205 µm; J. Prolab¯, Brazil), stored in microtubes and kept frozen at -18°C until further use (9).

### Liquid chromatography-electrospray ionization/multi-stage mass spectrometry (LC-ESI-MS)

LC-ESI-MS was used to confirm the presence of matesaponins, polyphenols and methylxanthines in first, second, fifth, tenth and fifteenth mates (extracts). LC analyses were conducted using an UHPLC Shimadzu device (Japan) equipped with a CBM-20A controller, LC-20AD pump and SIL 20AHT auto sampler. A Zorbax (Hungary) XDB-C8 column (150×4.6 mm, 5 µm) was used. The mobile phase consisted of water with 0.1% formic acid (A) and acetonitrile with 0.1% formic acid (B) at a flow rate of 0.3 mL/min according to the following gradient: 0.01–1 min, 90% solvent A; 1.01–4 min, 90–65% solvent A; 4.01–7 min, 65% solvent A; 7.01–11 min, 65–50% solvent A; 11.01–14 min, 50% solvent A; 14.01–17 min, 50–10% solvent A; 17.01–21 min, 10% solvent A; 21.01–23 min, 10–90% solvent A, and 23.01–30 min, 90% solvent A. The injection volume was 10 µL and the analysis was performed at 20°C. The mobile phase was prepared daily, filtered through a 0.45-µm membrane filter (Millipore, USA) and sonicated before use. The MS analyses were performed on a micrOTOF-QII (Bruker¯ Scientific, USA) with an electrospray ionization interface (ESI). TOF control data acquisition software was used. LC-ESI-MS was conducted in the positive-ion mode and operated under the following conditions: nitrogen gas temperature of 200°C, drying gas flow rate of 8 L/min, capillary voltage of 4000 eV, and ionization energy of 3 eV. Mass spectra were recorded in the full scan mode in the m/z range 50–1400.

### Fly strains and culture conditions

Wild-type *D. melanogaster* Harwich strains were obtained from the National Species Stock Center (USA). Flies were grown in the Clinical Analysis Laboratory (LEAC) from Universidade da Região da Campanha, RS, Brazil, where they were maintained on cornmeal medium at 25°C with a 12-h light/dark cycle.

### Diets

The standard diet (SD) used to keep the stocks available was prepared according to previously described formulation by Bahadorani et al. (13), with modifications. Briefly, 1 L of the standard diet consisted of 750 mL water, 37.5 g dry yeast, 7.5 g agar, 138 g corn flour, 65 g crystal sugar, 0.0038 g nipazol, and 3.5 mL acid solution (10 mL phosphoric acid and 100 mL acetic acid) in a sealed chamber. In order to obtain flies grown from eggs exposed to a high cholesterol diet, 1% synthetic cholesterol (3β-hydroxy-5-cholestene, 5-cholesten-3β-ol, Sigma Aldrich, USA) was added to the standard diet. The diet enriched with synthetic cholesterol was named the experimental diet (ED). To generate the stocks, 80 mL of the cooked mixture was poured into each vial.

For the fat-induced damage experiments, the synthetic cholesterol was added into the SD at 1% on a weight basis. Because synthetic cholesterol is insoluble in water, it was dissolved in dimethyl sulfoxide (DMSO). Synthetic cholesterol was added to 100 mL of DMSO (10 mM) and 5 mL of this solution was added to 1000 g of food.

### Exposure to yerba mate extracts

**Figure 1.**
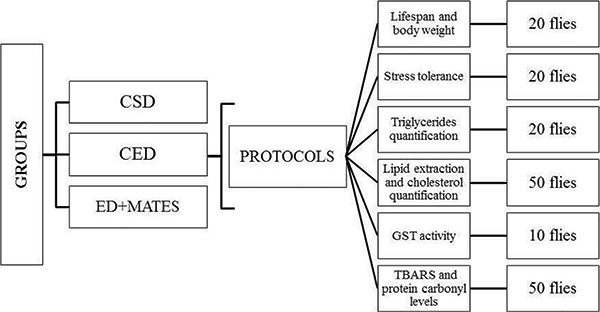
Flow diagram of the trial design. All experiments were performed in triplicate. CSD: control standard diet; CED: control experimental diet; ED+mates: experimental diet with yerba mates extracts; GST: glutathione S-transferases; TABRS: thiobarbituric acid reactive species.

Male flies (2–3 days old) were divided into different groups and were reared in vials; 30 mL of the cooked mixture was poured in each vial. In this study, only male flies were used because there is less hormonal effect in the male than in the female flies. All experimental protocols were performed in triplicate and had two control groups: one with the SD was named the control standard diet (CSD), and another one with the ED was named the control experimental diet (CED). In the ED+mate, the 1st, 2nd, 5th, 10th, and 15th mate extract fractions (1 mL) were added into the warm food and mixed with a small spatula. In the control groups, we used water instead of mate. The number of flies varied according to the protocols, as shown in Figure 1.

### Lifespan and body weight

To evaluate the effects of yerba mate extracts on the fly lifespan and body weight, we used a protocol that was previously described by Peng et al. (14) with minor modifications. Each group of 20 male flies was exposed to ED with the 1st, 2nd, 5th, 10th, and 15th mates (1 mL of each extract). The controls (CED and CSD) received water in the same volume. Dead flies were counted every 2–3 days, and the remaining flies were transferred to a new vial containing the same diet. Feeding lasted for 43 days.

The change in the average body weight per fly was used as an indicator of whether yerba mate extracts affected the food intake of the fruit flies. Flies in each vial were anesthetized with ether (diethyl ether ACS reagent) and, on the same days in which diet vials were changed, they were weighed in an analytical balance (BEL¯, Italy). The same insects that were subjected to the lifespan protocol were part of this assay. The average body weight per fly in each group was recorded. To confirm food consumption, we used the dye method in accordance with the protocol described by Verspoor et al. (15).

### Stress tolerance

#### Paraquat and hydrogen peroxide (H_2_O_2_) treatments

To examine the resistance of flies to stress induced by Paraquat (1,10-dimethyl-4,40-bi-pyridinium dichloride; Pq2+) or H_2_O_2_, we used the protocols proposed by Peng et al. (14) with minor modifications. Flies (n=20 per vials) were maintained on either the standard diet or experimental diet containing mate extracts. All flies were raised in 25°C. On day 10, fruit flies were first starved for 2 h and then transferred to new vials containing a filter paper saturated with 1 mL of 20 Mm paraquat that was diluted in a 6% glucose solution or 1 mL of 30% H_2_O_2_ diluted in 6% glucose. The number of dead flies was counted every 4–6 h until all flies were dead.

#### Cold and starvation resistance tests

The cold and starvation resistance tests were based on the method previously described by Heinrichsen and Haddad (16). In short, for cold resistance, a -5°C bath was made using water, ice and salt. Each group of 20 flies was placed in empty plastic vials and into the water bath. They remained as such for 2 h while the temperature was regularly checked throughout. At the end of the 2-h period, vials were removed from the water bath and flies were transferred to regular food and left to recover at room temperature. After 24 h, survival was recorded as the number of flies that had regained consciousness.

To analyze starvation, each group of 20 flies was placed in a plastic vial without food. A small, circular filter paper was placed in the bottom of the vial with 75 µL of water to prevent dehydration, and the water was replenished every 16 h or as needed. Survival was recorded every 4–8 h in each vial.

### Metabolic parameters and enzymatic activity

The metabolic determinations and enzymatic activity were evaluated from homogenized pools of animals. Pools of 10 flies were used to measure the enzymatic activity, 20 flies to evaluate triglycerides, and 50 flies to evaluate the total lipid extraction and cholesterol quantification. All determinations were performed three times with groups treated in the same conditions.

In these experiments, immediately after removing the flies from the treatment vials, they were frozen in liquid nitrogen and then rinsed with 1 mL of cold PBS to remove all traces of food that might be attached to the body (17).

#### Cholesterol

To quantify the cholesterol levels, we extracted the total lipids from fly samples. For this purpose, we used a pool of 50 flies that were homogenized in 1250 µL of 50 mM potassium phosphate buffer (TFK). The homogenate underwent extraction in a chloroform-methanol-water solution according to the lipid extraction method described by Bligh and Dyer (18).

Cholesterol was measured using the Cholesterol-liquiform Labtest kit¯ (Labtest, Brazil). This kit uses cholesterol oxidase to convert free cholesterol to cholest-4-en-one and hydrogen peroxide. Phenol and 4-aminoantipyrine are oxidized yielding quinoneimine, which has the maximum absorbance at 500 nm. The results are reported as mg/dL of tissue.

#### Triglycerides

The homogenate was centrifuged at 3000 *g* at 4°C for 10 min, and the supernatant was then aliquoted. Triglycerides were measured according to the reactions of lipase, glycerokinase, 1-P-glycerol oxidase, and peroxidase enzymes using the Triglycerides Liquiform-Labtest kit¯ (Labtest). The results are reported as mg/dL of tissue.

Since Tennessen et al. (17) observed that the presence of eye pigment in adult samples could interfere with accurate absorbance measurements at certain wavelengths, we tested the assay, before the analysis, at 505 nm using flies with or without heads and observed that the eye pigment did not have an effect at this wavelength.

#### Glutathione S-transferase

The glutathione S-transferase (GST) activity was measured as described by Habig and Jakoby (19) using 1-chloro-2,4-dinitrobenzene (CDNB) as a substrate. One unit of GST was calculated as µmol of CDNB conjugate per mg protein (U/mg protein), using the molar extinction coefficient of 9.6 mM/cm. The enzymatic activity was expressed as GST units/dL protein.

### Thiobarbituric acid reactive species (TBARS) levels

Lipid peroxidation was assayed according to a method proposed by Ohkawa et al. (20). Briefly, fly homogenate (1:20 w/v) was mixed in a medium containing 8.1% sodium dodecyl sulfate, acetic acid buffer, pH 3.5, and 0.8% aqueous solution of thiobarbituric acid. After heating at 95°C for 120 min, the red pigment produced was spectrophotometrically measured at 532 nm. The results were calculated using a standard curve of malondialdehyde (MDA) and corrected by tissue milligrams. The results are expressed as nanomoles of MDA per milligram of tissue.

### Protein carbonyl levels

Protein carbonyl was measured according Levine et al. (21) with some modifications. Fly homogenates were derivatized using 2,4-dinitrophenylhydrazine (DNPH). DNPH reaction proteins were precipitated with an equal volume of 20% trichloroacetic acid and, after centrifugation (15,000 *g*, 15 min, 4°C), they were washed three times with an ethanol/ethyl acetate mixture (1:1). Finally, the precipitates were dissolved in 2% sodium dodecyl sulfate. Protein carbonyl levels were spectrophotometrically determined at 370 nm, compared to blanks. The results were calculated using the molar extinction coefficient of DNPH, which was corrected by the protein content and reported as nanomoles of carbonyl per milligram of protein.

### Statistical analysis

Data are reported as means±SD. For survival and longevity analysis, we performed a dose-response curve and using nonlinear regression followed by the Shapiro-Wilk and Logrank tests. All other data were subjected to one-way ANOVA followed by the Dunnett's test. Differences between groups were considered significant at P≤0.05.

## Results

The choice of herb used in this study was based on a previous report from our group. The Uruguayan brand Baldo¯ presented the highest content of polyphenols and methylxanthines. Moreover, compared with herbs from Argentina and Brazil, this brand has the most effective antioxidant capacity (9,10).

In addition, in the quoted study was observed that all compounds examined showed a similar decrease in concentration over subsequent extractions. The concentrations of the compounds analyzed by HPLC analysis was quantified as follows: chlorogenic acid > caffeic acid > caffeine > theobromine (9).

### LC-ESI-MS

**Figure 2.**
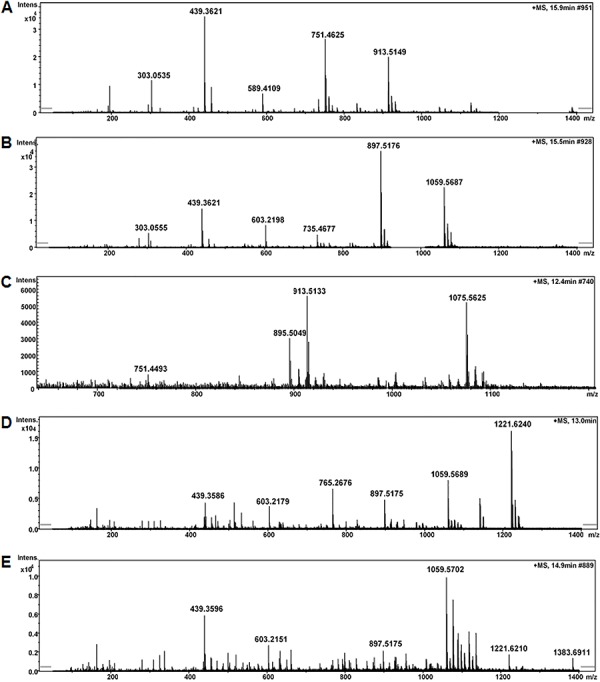
Spectra of ion fragments obtained from analysis of yerba mate (*Ilex paraguariensis*) extracts. Analysis performed by using electrospray ionization in positive ion mode. Ion fragments correspond to chemical compounds presented in Table 1. *A*, Matesaponin 1 ([M+H]+=913); *B*, Matesaponin 2 ([M+H]+=1059); *C*, Matesaponin 3 ([M+H]+=1075); *D*, Matesaponin 4 ([M+H]+=1221); *E*, Matesaponin 5 ([M+H]+=1383).

**Figure 3.**
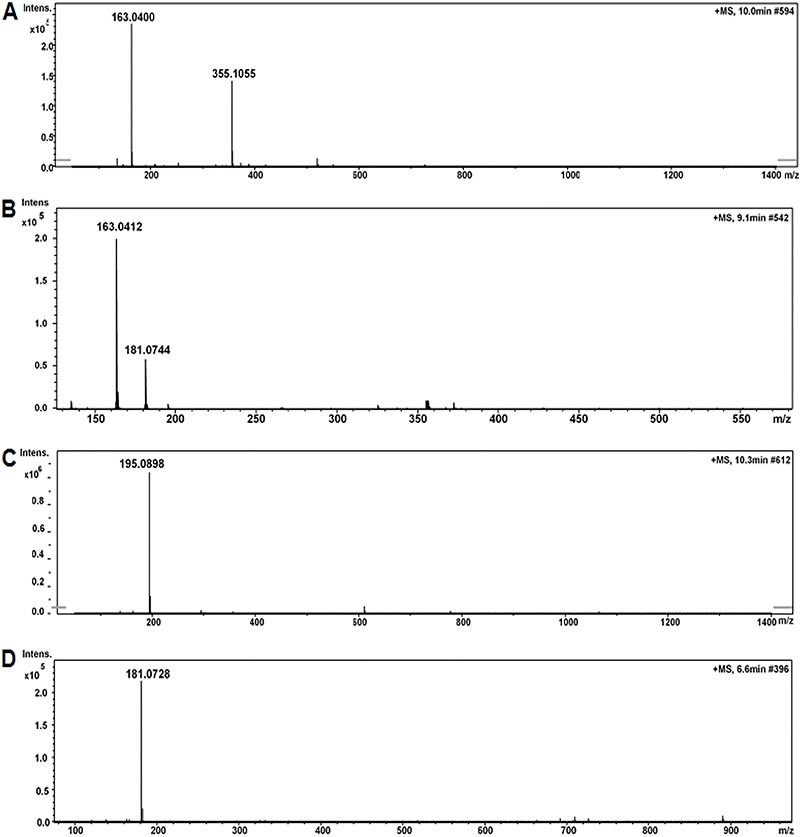
Spectra of ion fragments obtained from analysis of yerba mate (*Ilex paraguariensis*) extracts. Analysis performed by using electrospray ionization in positive ion mode. Ion fragments correspond to chemical compounds presented in Table 1. *A*, Chlorogenic acid ([M+H]+=355); *B*, Caffeic acid ([M+H]+=182); *C*, Caffeine ([M+H]+=195); *D*, Theobromine ([M+H]+=181).

Aqueous *I. paraguariensis* extract, prepared as mate or chimarrão beverage, was monitored by HPLC-DAD and submitted to ESI-MS analyses. Standards, UV spectra, MS+ fragmentation and previous reports confirmed the presence of matesaponins, phenolic compounds and methylxanthines in all samples. Table 1 lists the identified compounds, their retention times (Rt), molecular ions [M+H]+ and fragment ions, whereas Figures 2 and 3 illustrate the mass spectra. It is remarkable that all the nine components identified were detected during the successive extractions, being present in all mate tested.

**Table 1.** Chemical components identified in yerba mate extract with corresponding retention times (Rt), quasi-molecular ions in the positive mode and key fragments LC-ESI-MS.

Compound	Rt (min)	Ion [M+H]+	MS^+^ fragmentation (m/z)
Matesaponin 1	15.9	913	751[M+H-Glc]; 589[M+H- 2Glc]; 439[M+H- 2Glc-Ara]
Matesaponin 2	15.5	1059	897[M+H-Glc]; 735[M+H-Glc-Rha], 603[M+H-Glc-Rha-Ara]; 439 [M+H- 2Glc-Rha-Ara]
Matesaponin 3	12.4	1075	913 [M+H-Glc]; 751[M+H-2Glc]; 439[M+H- 3Glc-Ara]
Matesaponin 4	13.0	1221	1059[M+H-Glc]; 897[M+H-Glc-Rha]; 765[M+H-Glc-Rha-Ara]; 603[M+H-2Glc-Rha-Ara]; 439[M+H- 3Glc-Rha-Ara]
Matesaponin 5	14.9	1383	1221[M+H-Glc]; 1059[M+H-Glc-Rha]; 603[M+H-2Gluc-Rha-Ara]; 439[M+H- 4Glc-Rha-Ara]
Chlorogenic acid	10.0	355	163 [M+H-195]
Caffeic acid	9.1	182	163 [M+H-H2O]
Caffeine	10.3	195	FND
Theobromine	6.6	181	FND

LC-ESI-MS: liquid chromatography-electrospray ionization/multi-stage mass spectrometry; Glc: glucose; Rha: rhamnose; Ara: arabinose; FND: fragments not detected.

### Lifespan and body weight

**Figure 4.**
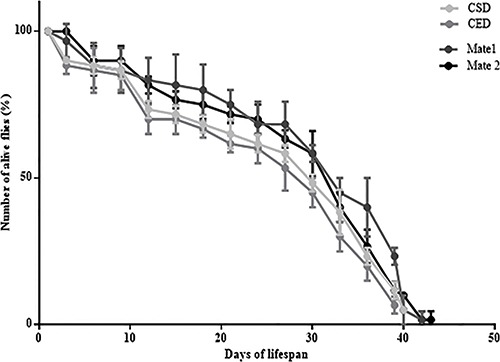
Lifespan curve of flies fed on a control standard diet (CSD), control experimental diet (CED) and experimental diet containing yerba mates 1 and 2 (1 mL of extracts). The trials were made in triplicate and the data are reported as the maximum lifespan of the last fly and mean±SD lifespan for each group (there were 20 flies in each group). P<0.05, the Shapiro Wilk test showed that mates 1 and 2 (black plots) could significantly extend the mean lifespan of fruit flies compared to CSD and CED (gray plots).

Herein, fruit flies exposed to a high cholesterol diet were treated with *yerba mate* extracts. The corresponding analyses revealed that mate treatment increased lifespan. The maximum lifespans for the mate 1 and 2 treated groups were 42 and 43 days, respectively, compared with 38 days for flies on the experimental diet and 40 days for flies on the standard diet. There was no statistically significant difference between the controls and other extracts (mates). Figure 4 presents the longevity curve in the groups where there was statistical significance.

No significant difference in body weight was observed between the two controls and yerba mate extract-treated groups. The diet consumption in all experimental groups has been confirmed by the intestinal flies' coloration (blue dye, Supplementary Figure S1).

### Stress tolerance

**Figure 5.**
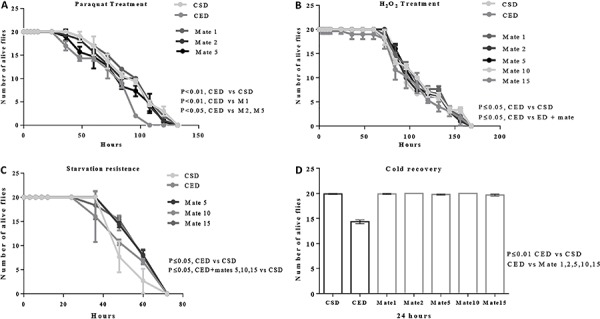
Experimental paradigm for flies' resistance to paraquat treatment (*A*), hydrogen peroxide treatment (*B*), starvation (*C*) and cold recovery (*D*). Flies were placed on control standard diet (CSD), control experimental diet (CED), or experimental diet+mates (M1, M2, M5, M10, M15) for 10 days. They were then submitted to the tests (Shapiro-Wilk and ANOVA followed by the Dunnett's test).

The results from the paraquat challenge test showed that flies exposed to the CED had less resistance (P<0.01) than those exposed to the CSD. When the groups treated with mates were compared to the CED, we confirmed that the resistance was significantly improved in flies receiving mates 1, 2 and 5 (P<0.01). The maximum survival time was 120 h in flies exposed to the CSD, and for the groups treated with mates 1 and 2. The minimum survival time was 96 h for the CED and mate 15-treated group (Figure 5A).

The insects were also susceptible to damage produced by H_2_O_2_ exposure. The CED fly groups had a lower capacity to resist to this free radical species when they were compared to the CSD group and ED+mate-treated group (P<0.05). There was no significant difference between the ED+mate and CSD (Figure 5B).

With respect to the capacity to resist starvation conditions, flies consuming diets with cholesterol supplementation were more resistant to starvation than flies receiving standard diet. After 48 h of starvation, 80% of flies in the CSD died, in contrast to 55% of flies in the CED and 35% of flies in the ED+mates (Figure 5C). The starvation resistance was significantly higher in flies fed CED and ED+mates compared to the CSD (P<0.05). Moreover, the data showed that flies in ED did not completely recover after 2 h of cold exposure (Figure 5D).

### Metabolic parameters

**Figure 6.**
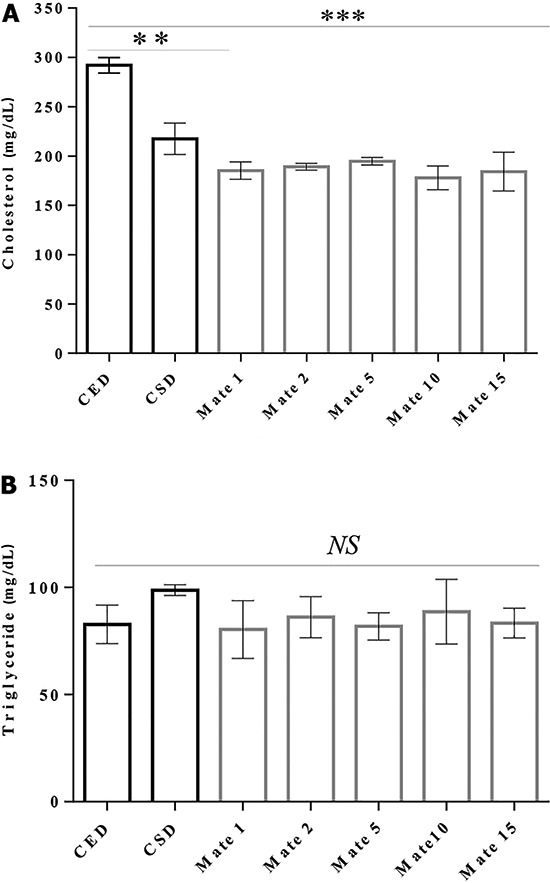
Cholesterol (*A*) and triglyceride (*B*) levels (per mg live weight) in the whole-body homogenate of flies after 10 days on various diets: standard (CSD), experimental (CED) and experimental diet+mates (Mate 1 to Mate 15). Flies were homogenized in groups of 20 males. Data are reported as means±SE. NS: not significant. **P<0.01, ***P<0.001, one-way ANOVA followed by Dunnett's multiple comparisons test.

To quantify the flies' cholesterol levels, we extracted the total lipids from samples. Our results showed that there were changes in the cholesterol levels in flies with different treatments (Figure 6A). Comparing the cholesterol levels in the CSD (217.4±15.8 mg/dL) and CED (291.9±7.8 mg/dL), we found a significant increase in the cholesterol levels in flies that were fed with an experimental diet (P<0.05). Flies given ED+mate (all groups tested) presented with lower cholesterol levels than those given ED alone (P<0.01). In terms of triglyceride (TAG), no significant difference was observed in the tested treatments (Figure 6B).

### GST activity

**Figure 7.**
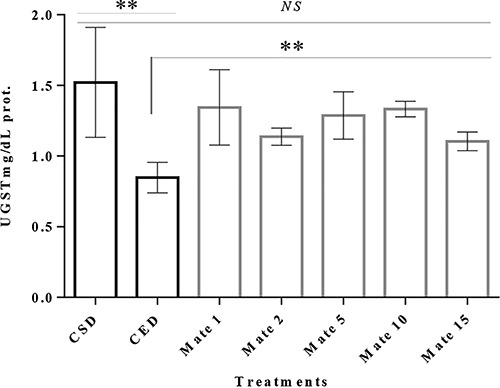
Glutathione S-transferases (GST) activity in the whole-body homogenate of flies after 10 days on various diets: control standard diet (CSD), control experimental diet (CED) and experimental diet+mates (Mate 1 to Mate 15). Flies were homogenized in groups of 20 males. NS: not significant. Data are reported as means±SE. **P<0.01, ANOVA followed by Dunnett's test.

GST activity was reduced in a pool of flies in the CED compared to the CSD and ED+mates. It was possible to observe that the yerba mate extracts increased their activity in a significant manner (P<0.01; Figure 7).

### TBARS and protein carbonyl levels

**Figure 8.**
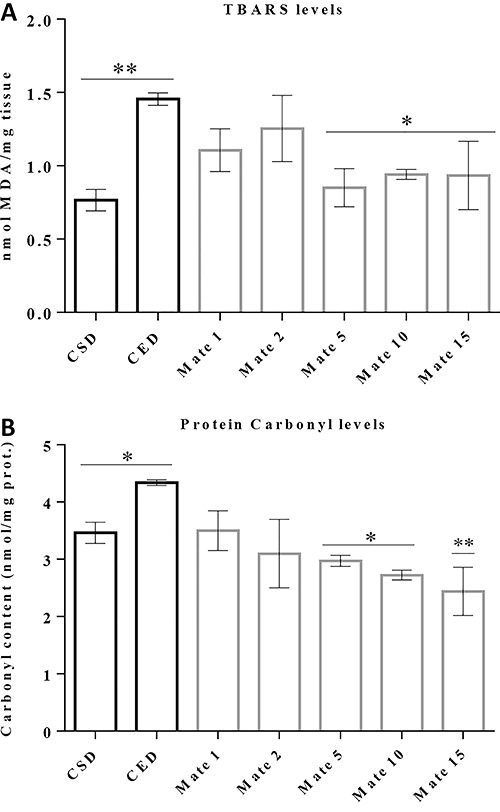
TBARS and carbonyl protein levels. Flies were homogenized in groups of 50 males after 10 days on various diets: control standard diet (CSD), control experimental diet (CED) and experimental diet+mates (Mate 1 to Mate 15). Data are reported as means±SE for n=3. *P<0.05, **P<0.01, ANOVA followed by Dunnett's test.

Lipid peroxidation and protein carbonylation are induced by a high cholesterol diet, which can be reduced by the *yerba mate* extracts. Our data showed an increase in TBARS and protein carbonylation level in the CED group compared to the CSD (P<0.05). In these two experiments, mates effectively reduced the malondialdehyde and carbonyl levels (reactive intermediates from lipid peroxidation and protein carbonylation) at standard diet (CSD) level (Figure 8A and B). Results showed that mates 5, 10 and 15 were more effective to reduce the TBARS and protein carbonylation levels.

## Discussion

The research describes an investigation about the effect of yerba mate on the lifespan of *D. melanogaster*. First, we evaluated the extracts to confirm the presence of the target compounds. A previous study by our group described some phenolic compounds and methylxanthines from yerba mate (10). Here, we also detected five matesaponins in the extract. According to the literature, matesaponins in *I. paraguariensis* samples mainly consist of ursolic acid derivatives (22).

Matesaponin 1, which had a molecular ion *m/z* of 913.51 [M+H]+ with two losses of 162 Da and one loss of 150 Da, corresponding to two glycosyl and one arabinosyl residues (23). Fragmentation associated with loss of sugar molecules is common, because matesaponins are glycosylated compounds. Previous studies mainly report the presence of glucose, arabinose and rhaminose sugars linked to *I. paraguariensis* saponins (22,24,25).

In sequence, the ion at m/z 1059.57 [M+H]+ for matesaponin 2 gave rise to fragments that are consistent with losses of four sugar moieties, which we identified as 2Glc-Rha-Ara, resulting in *m/z* 439 (ursolic acid aglycone).

Matesaponin 3 (*m/z* 1075.56) has fragments at 913, 751 and 439, which have corresponding losses of four saccharide units (3Glc-Ara). Matesaponin 4 gave an ion at *m/z* 1221 [M+H]+ with fragments at *m/z* 1059, 897, 765, 603 and 439, confirming the loss of (Glc-Rha-Ara-Glc-Glc-Glc) as a linear sequence and resulting in aglycone. The matesaponin 5 structure had a molecular ion at *m/z* 1383 with losses of six sugar moieties, which is consistent with a multiple glycosylated ursolic acid ester (22).

Positive ESI-MS also confirmed the presence of ions [M+H]+, chlorogenic acid (*m/z* 353), caffeic acid (*m/z* 181), caffeine (*m/z* 195) and theobromine (*m/z* 181), as seen in Table 1. Although these compounds have not generated fragments, previous HPLC-DAD analysis, with standards, confirmed their presence (9).

Previously, we had reported that the yerba mate extracts had significant antioxidant capacity and could increase the longevity and survival of *Caenorhabditis elegans* (9,10). Our hypothesis was that these extracts contain compounds that could target multiple molecular pathways to produce protection against oxidative stress, and that this may be important in ability of mate to extend lifespan. Other studies also demonstrated that extracts obtained from natural foods increase the *D. melanogaster* lifetime. Blueberry extracts prolong the mean lifespan of fruit flies by 10% (1); also, black tea improves the survival time of fruit flies (14). Wang et al. (5) demonstrated that cranberry anthocyanin extract extended the *D. melanogaster* lifespan and observed that this activity was directly attributable to its antioxidant activity after absorption.

Despite the fact that the molecular mechanism by which polyphenol compounds extend lifespan is not entirely known, it is known that a high-fat diet can induce higher levels of ROS, as evidenced by hydrogen peroxide (H_2_O_2_) emission from the mitochondria. This indicates that oxidative phosphorylation is more active, requiring more reducing equivalents (4). Moreover, a fat diet increases the iron absorption and affects its regulation and use (26). This is relevant because iron plays a central role generating free radicals. In fact, plant extracts can chelate Fe2+ and reduce its availability for interacting with H_2_O_2_, which decreases the hydroxyl radical formation via the Fenton reaction (27).

Additionally, there is growing evidence that phytochemicals prolong the lifespan by modulating the network signaling pathways (28). Lima et al. (10) suggested that the effect in longevity observed in *C. elegans* treated with yerba mate extracts was linked to a decrease in ROS and an increase in the DAF-16 migration into the cell nucleus, which is probably due to the interaction between molecular pathways in the presence of high polyphenols levels.

In addition, flies with higher cholesterol levels were less capable of responding to free radicals. This occurs probably because dietary fats modulate changes in the lipids and can initiate a sequence of events that lead to cell damage. Thus, failing to keep H_2_O_2_ low promotes considerable toxicity due to the production of highly reactive species, such as hydroxyl radicals (29). In our study, yerba mate extracts delayed the harmful cycle. Phenolic compounds can donate an electron to O_2•_- (e.g.), which was accompanied by a proton-transfer process to produce a phenoxyl radical (30), which is more stable and less reactive.

It was also observed that the flies submitted to ED+ mates were more resistant to starvation conditions, and that flies fed in ED were unable to recover after exposure to severe cold. Since metabolic adaptations are important to survival, these responses can be associated first to the lipids stored within the body fat that are used as a vital resource to survive through periods of low nutrient availability (31). Flies given a high-fat diet are unable to activate some proteins and genes, jeopardizing the cell membrane integrity and impairing the ability to recover from cold stress (16). The role of the compounds present in extracts could be related to fat oxidation control that ensures the thermogenesis, facilitating the regulation of fat mobilization and reducing lipid peroxidation level, which would also increase the cell membrane resistance.

Hypercholesterolemia is associated with the deterioration of antioxidant status, resulting from increased MDA levels (32). In addition, a high-fat diet causes cholesterol accumulation, and induce hyperglycemia and insulin resistance, two alterations that are linked to oxidative stress. Hydrogen peroxide impairs insulin signaling and inhibits glucose transport (26). We propose that reducing the levels of cholesterol in male flies fed with an experimental diet supplemented with yerba mate extracts, can be attributable to an association between antioxidant effects and enzymatic/transcriptional mechanisms, which regulate and modulate DHR96 expression. The DHR96 nuclear receptor plays an essential role in coordinating the TAG, cholesterol breakdown, absorption and trafficking (33). The increase in the cholesterol levels was not sufficient to promote deposits in flies' tissues. The average weight did not vary substantially, which is in accordance with this result. This effect can also be related to the DHR96 regulation. Future experiments in flies with elevated or decreased DHR96 expression levels will help to inform us on the role of the extracts in these parameters.

The DHR96 nuclear receptor also regulates the xenobiotic responses in *Drosophila* (33). There are several possible mechanisms by which the metabolic functions of DHR96 could affect detoxification responses, both in the exchange of dietary nutrients as well as in the coordination of xenobiotic and metabolic responses within the animal (34). Excess fat consumption plays a crucial role in activating high-fat diet modulated lipid metabolic pathways and can deregulate the glutathione levels (35). The results described by Curtis et al. (36), which focus on the role of GSTA4, suggest that the GSTA4 downregulation increases the protein carbonylation and alters the glucose and lipid metabolism.

Although we have not assessed the ROS levels, increased TBARS and carbonyl levels can be used as an indirect evidence of high ROS production (37). Considering that lipid peroxidation is a self-propagating autocatalytic process producing several potent ROS (3), and that the fatty acid composition of membranes might be important in aging processes, our data support the role of antioxidants in protecting lipid oxidative damage and the function of GST in the detoxification process against lipid peroxidation derived products (38).

We believe that extracts' effects are optimal even in low compound concentrations, which agrees with the definition that antioxidant is a substance that, when present at low concentrations compared to an oxidizable compound, delay or prevent oxidative damage caused by the presence of ROS. Moreover, it is essential to consider that extracts of natural products are complex mixtures of different bioactive compounds that may act synergistically to determine its effects (39). The antioxidant intervention, based on compounds that act as free radical scavengers to detoxify oxidative-derived carbonyl reaction products, represents a new therapeutic target to trap the lipid-derived reactive carbonyl species (40).

In conclusion, yerba mate extracts in the diet could prolong the mean lifespan, decrease induced mortality and recover the enzymatic detoxification functions in fruit flies. The antiaging activity and antioxidant effects of yerba mate were associated mainly with the compounds in extracts. Despite the present observations that reveal the beneficial properties of *I. paraguariensis* beverages, energy metabolism in flies is likely associated with complex mechanisms in which the diet can positively or negatively affect the events involved in these interactions. Future studies should address the issue of bioavailability and metabolism of matesaponins, phenolic compounds and methylxanthines in fruit flies.

## Supplementary material

Click here to view [pdf].
